# The multiple realization of human color vision revisited

**DOI:** 10.3389/fpsyg.2022.985267

**Published:** 2022-09-20

**Authors:** Ken Aizawa

**Affiliations:** Department of Philosophy, Rutgers University, Newark, NJ, United States

**Keywords:** realization, multiple realization, Dimensioned realization, human color vision, trichromacy

## Abstract

Over the last 25 years, there has been a concerted effort to settle questions about multiple realization by bringing detailed scientific evidence to bear. Ken Aizawa and Carl Gillett have pursued this scientific approach to multiple realization with a precise theory and applications. This paper reviews the application of the Dimensioned approach to human color vision, addressing objections that have appeared in the literature.

Over the last 25 years, there has been a concerted effort to settle questions about multiple realization by bringing detailed scientific evidence to bear. Bechtel and Mundale (1999), proposed that scientific work on human brain mapping presented a challenge to multiple realization.^1^ Bickle (2003) proposed that the biochemistry of memory consolidation presented a challenge to multiple realization.^2^ Weiskopf (2011) proposed that the visual systems of *Limulus polyphemus* illustrate multiple realization. Many others have investigated multiple realization in the context of evolution by natural selection.^3^ And even these examples do not exhaust this approach.^4^

Some philosophers of science have pursued this scientific approach to multiple realization with a precise theory and applications. On the theoretical side, Gillett (2002, 2003) proposed a Dimensioned view of realization. Aizawa and Gillett, 2009a,b, added a complementary theory of Dimensioned multiple realization. On the applied side, Aizawa and Gillett have considered a number of examples from neurobiology, the most detailed of which concerns human color vision. Aizawa and Gillett (2009a,2011) and Aizawa (2013, 2020), proposed that normal human color vision is multiply realized by distinct sets of property instances of the absorption spectra of retinal cone opsins. Aizawa and Gillett (2011) also proposed that normal color vision is multiply realized by distinct sets of property instances of proteins, such as transducin, in the phototransduction biochemical cascade.

Corresponding to the two-fold character of the Aizawa-Gillett project, one can object to both the theory and its applications. One might argue that the theoretical account does not correctly characterize the compositional relations in science that it is meant to characterize, or one can argue that the examples do not fit the theory.

Polger and Shapiro (2016), presses both kinds of objection. It rejects the Aizawa-Gillett theories of realization and multiple realization and the Aizawa-Gillett conclusion that human trichromatic vision is multiply realized. Against the Aizawa-Gillett account of multiple realization, they claim that “philosophers like Ken Aizawa and Carl Gillett … who allow variation of any sort to distinguish between realizations—as little as a difference of a single molecule—are heading down the wrong path” [Polger and Shapiro (2016), p. 62]. Concerning color vision, they write, “the example of variations in human cone opsins does not make for concrete direct evidence of actual multiple realization of a psychological capacity” [Polger and Shapiro (2016), p. 110].

Balari and Lorenzo (2019) offer a bold criticism of Aizawa and Gillett’s applied work. In a section of their paper labeled “The dismissal of scientific practice,” Balari and Lorenzo claim that Aizawa and Gillett “actually ignore scientific practice.” Although they aim their fire at Aizawa and Gillett’s handling of long-term potentiation (LTP), one can easily see how their concern would extend to the discussion of human color vision.

Strappini et al. (2020) make a case for the multiple realization of visual crowding in humans. This is an instance of what is sometimes described as ‘‘intraspecific multiple realization,’’ by which they mean a property that is multiply realized by members of a single biological species. For this case, they embrace the Aizawa-Gillett theory of multiple realization. Moreover, they noted the significance of human color vision as a potential case of intraspecific multiple realization. Despite their sympathies with the Aizawa-Gillett approach to multiple realization, however, they expressed various reservations one might have about concluding that human color vision as treated by scientists is multiply realized.^5^

Given the interest in the Aizawa-Gillett approach to multiple realization and the possible multiple realization of human color vision, it is important to address both theoretical and applied objections that have appeared in the literature. To this end, some guidance is needed about the sometimes complicated features of the Dimensioned framework for realization and multiple realization and their application to some of the relevant science of human color vision. The term “guidance” should be noted. The goal here is not to work once again through all the scientific and theoretical details that have been presented in earlier works (Gillett, 2002, 2003, 2013a,^2016^, Aizawa, 2007, 2018a,b,^2020^, Aizawa and Gillett, 2009a,^2011^, ^2019^—perhaps there is no need for *that*—but instead to highlight the principal points that would help interested readers to navigate those details.

Section “Realization and multiple realization: The theories” reviews the Aizawa-Gillett theory of Dimensioned realization and the complementary account of Dimensioned multiple realization. Section “An application of the theories: Human color vision” reviews the application of the theory to human color vision. Section “Critiques of the Aizawa-Gillett theories” will address Polger and Shapiro’s objections to the theories of realization and multiple realization. Section “Critiques of the Aizawa-Gillett application” addresses the multiple critiques of the application of the theory to human color vision.

## Realization and multiple realization: The theories

Dimensioned realization and multiple realization have been given extensive and detailed exposition in other works.^6^ The goals of the presentation here, therefore, are more focused. One goal is to provide a simple and accessible presentation of the view, setting aside various details. The second goal is to highlight features of the theory that address objections.

The core idea of Dimensioned Realization is that the relations between properties in scientific explanations are often a species of many-one compositional determination relation—one type of ontological determination relation.^7^ Consider an extremely simple example, the dipole moment of a molecule of hydrogen fluoride (HF). The dipole moment of a molecule is its “charge imbalance.” A HF molecule is more negative on the fluorine side of the molecule than it is on the hydrogen side. The standard scientific explanation of this charge asymmetry is that fluorine is more electronegative than is hydrogen, so that electrons tend to cluster closer to it than to the hydrogen. This makes the fluorine side more negative. Thus, we have a scientific explanation of a property instance of a whole—the dipole moment of a molecule—in terms of property instances of its constituent parts—the electronegativities of its constituent atoms. This example is about as theoretically simple as the Dimensioned view allows.

A more precise account of the matter requires certain complications. First, one wants a theory of the property instances involved. To this end, Aizawa and Gillett rely upon a version of the causal theory of properties according to which properties confer powers upon individuals. Second, property instances stand in this relation only under certain background conditions, the most familiar of which are temperature and pressure. To give an example, a given cone opsin molecule will have a specific absorption spectrum only under a limited range of temperatures. Above a certain temperature, the protein changes its conformational structure, thereby changing its sensitivity to different frequencies of light.

Setting aside many important features, we get this precise schema for a “Dimensioned” account of realization:

Property/relation instance(s) F_1_-F_n_ realize an instance of a property G, in an individual *s* under conditions $, *if and only if*, under $, F_1_-F_n_ together contribute powers, to *s* or *s*’s part(s)/constituent(s), in virtue of which *s* has powers that are individuative of an instance of G, but not vice versa (Aizawa and Gillett, 2011, p. 202).

In this schema, the inclusion of the contribution of powers reflects the commitment to the causal theory of properties.^8^ Further, the reference to conditions $ reflects the acknowledgment of the role of background conditions.^9^

The core idea of Dimensioned multiple realization is that one must have one set of property instances F_1_–F_n_ in certain parts that realizes an instance of G in some whole and another non-identical set of property instances F*_1_ –F*_m_ that realizes an instance of G, where the parts and whole may be different. Take an example selected for simplicity. Both H_2_ and O_2_ have no dipole moments. This is because the electronegativity of one atom in the molecule is that same as the electronegativity of the other atom in the molecule. The electronegativity of one atom balances the electronegativity of another. It is easy to see how one might generalize this. Dipole moments are vector quantities. They are directional magnitudes. Any two molecules that have the same vector sum of dipoles among their constituent chemical bonds will have the same dipole moment. Benzene, with its planar symmetric structure, also lacks a dipole moment. The explanans for the dipole moment of benzene will be different than the explanans for the dipole moment of O_2_.

Commentators have sometimes objected to the example of dipole moments. Why, one might ask, do we need the complicated schema Aizawa and Gillett offer (see below) in order to understand such a simple bit of science as the dipole moment? The answer is that the theory is not meant to illuminate the dipole moment; instead, the dipole moment is supposed to illuminate the schema. Indeed, there is one realized property of having no dipole moment that molecules of both H_2_ and O_2_ both have. Further, the electronegativities of H and O are different. There is, thus, one realized property and two different realizer properties. This is about as simple as it can get. Examples from psychology are likely to be much more complicated as many more properties will be involved. Further, the example is far from contested territory in cognitive science. Further, the example is scientific and one can, in fact, fit the example into the proposed schema.^10^

The core idea of Dimensioned multiple realization is that two sets of property instances F_1_–F_n_ and F*_1_ –F*_m_ realize instances of G. Matters are not, however, that simple. One does not count the realization of, say, pain at the neuronal level and at the biochemical level as multiple realizations of pain. One wants the distinct realizers of G to be at the same scientific level. All of this is captured in the following schema:

A property G is multiply realized if and only if (i) under condition $, an individual s has an instance of property G in virtue of the powers contributed by instances of properties/relations F_1_–F_n_ to s, or s’s constituents, but not vice versa; (ii) under condition $* (which may or may not be identical to $), an individual s* (which may or may not be identical to s) has an instance of property G in virtue of the powers contributed by instances of properties/relations F*_1_ –F*_m_ of s* or s*’s constituents, but not vice versa; (iii) F_1_–F_n_ ≠ F*_1_–F*_m_ and (iv), under conditions $ and $*, F_1_–F_n_ of s and F*_1_–F*_m_ of s* are at the same scientific level of properties.

Notice that, since realization is a many-one relation, multiple realization obtains when one set of property instances is distinct from another set of property instances.

## An application of the theories: Human color vision

Aizawa and Gillett aspire to providing an account of the compositional relations among properties that scientists postulate. It is an account of relations that scientists implicitly rely upon in providing compositional explanations. Given this goal, it is important to show that it applies to actual cases. The dipole moment cases show that. But we can show that it also applies to much more complicated cases in psychology, as in vision science.

Aizawa and Gillett choose an example from the science of human color vision as it is *prima facie* a case of Dimensioned realization and Dimensioned multiple realization. They suppose that individual humans have normal color vision and that individual humans have normal color vision in virtue of, among other things, the spectral sensitivities of some of their parts, namely, the opsins contained in retinal cones. The structure of the case is relatively simple. Scientists screen individuals for normal color vision using simple tests, such as the Ishihara test. Individuals making the correct identifications of numerals in the set of plates are deemed to have normal color vision. It is also possible to use genetic tests to determine, for example, whether an individual male has, say, Red(Ser^180^) versus Red(Ala^180^). (Since color vision is a sex-linked trait, we presuppose a male so that there is only one red cone opsin.) Biophysical measurements reveal that these distinct cone opsins—Red(Ser^180^) and Red(Ala^180^)—have distinct absorption spectra.^11^ Their peak sensitivities are somewhat different. Thus, we have the same property realized in the two males, but the two males have different realizers at the biochemical level. This is, in essence, Dimensioned multiple realization.

The preceding part of the story focuses on the cone opsins, but there is another part that focuses on subsequent steps in the biochemical phototransduction pathway.^12^ The discussion in Aizawa and Gillett (2011) is complicated, so a simpler presentation is in order. For present purposes, it suffices to focus on the second protein in the phototransduction pathway, a G-protein sometimes called ‘‘transducin.’’ Upon absorption of a photon, a single photopigment molecule will change conformation. After this conformational change, the molecule breaks into two components, a retinal chromophore and an opsin protein. The opsin component binds to a single transducin molecule. This transducin molecule, in turn, activates a molecule of an enzyme, cGMP phosphodiesterase. There are known genetic mutations to transducin.^13^ Such mutations are likely to give rise to differences in property instances in distinct transducin molecules that realize normal color vision, hence give rise to multiple realization. What is important, and underappreciated, about this example is that the differences in transducin properties do not induce individual differences in color discrimination. Differences in transducin property instances are causally downstream from the cone opsins that are differentially sensitive to the frequency of captured photons. If this analysis is correct, then the putative multiple realization of normal color vision cannot be dismissed on the grounds that differences among realizer property instances induce individual differences among those individuals bearing a multiply realized property.^14^

## Critiques of the Aizawa-Gillett theories

As noted in the introduction, critics have raised objections to both the theoretical component of the work and to its application. In this section, we begin with objections Polger and Shapiro have raised to the theoretical component of the project.

Aizawa and Gillett assess their account by how well it captures the relation implicit in certain compositional explanations in the sciences. Polger and Shapiro, however, have objected to Dimensioned Realization on a different basis:

An account of realization should discriminate between realization and other dependence relations—other ways that things can be made up.. Moreover, and again in contrast to Gillett, it is informative because it does not posit realization everywhere. Some things are realized, and some are not [Polger and Shapiro (2016), pp. 29–30, cf. p. 28, fn. 14.]

It is easy to see how one might have this objection, as there is no one place in Aizawa and Gillett’s works that specifically addresses it. One must, instead, survey a number of their works for the response to come into focus. The first piece of what has become the Aizawa-Gillett picture—the Dimensioned view of realization—was first broached in Gillett (2002, 2003). Gillett (2013a), adds to this a theory of scientific constitution as another dependence relation alongside realization. This is a theory of the dependence relation between an individual and its parts, as for example the relation between a cell and its organelles. Further, Gillett (2013b,2016) outlines a theory of implementation that characterizes the dependence relation between the activity of an individual, such as the contraction of a muscle, and the activities of its constituent parts, such as the binding of myosin to actin filaments, the hydrolysis of ATP, and the conformational change of myosin. Aizawa and Gillett (2019) propose that these distinct species of dependency relations figure into distinct species of compositional explanations. Thus, Polger and Shapiro’s contentions notwithstanding, Aizawa and Gillett do discriminate between realization and other dependence relations.

Moreover, Aizawa and Gillett do not posit realization everywhere. For one thing, some things (property instances) are realized, and other things (individuals and activities) are not. These other things are constituted or implemented. For another, Aizawa and Gillett do not think the property instances of microphysics have been shown to be realized (Gillett, 2016). They think that some property instances are realized (i.e., property instances of non-basic individuals of the special sciences) and some are not (i.e., property instances of basic individuals of basic physics).

Turn now to Polger and Shapiro’s criticism of the theory of multiple realization. They claim that “On some views, variation of any sort suffices for multiple realization” (Polger and Shapiro, 2016, p. 38) and suggest that Aizawa and Gillett have one of these views. This is not correct. Consider two individual cone opsins, Red(ala^180^), a “red” cone opsin with an alanine amino acid at position 180, and Red(ser^180^), a “red” cone opsin with a serine amino acid at position 180. These molecules differ in their absorption spectra. They also differ in their polarity, since serine has a hydroxyl group where alanine has only a proton. The differences in absorption spectra are relevant to the multiple realization of normal human color vision because the absorption spectra contribute powers that are individuative of the property of normal human color vision. By contrast, the differences in the polarity are not relevant to the multiple realization of normal human color vision, because the polarity does not contribute powers that are individuative of normal human color vision. Many other properties of Red(ser^180^) and Red(ala^180^), such as their shape, size, etc., would serve to make the same point.

Gillett (2003) made essentially this point informally even before the formulation of the schema for multiple realization. Gillett, first, proposes that “only properties/relations that result in the powers of the realized property are taken to be relevant to (multiple realization)” (Gillett, 2003, p. 598). As an example, Gillett proposes that the properties/relations of aluminum atoms in one corkscrew, label them F_1_–F_n_, provide one realization of the property of being a corkscrew, whereas the different properties/relations of steel atoms, label them F*_1_–F*_m_, provide a distinct realization of the property of being a corkscrew. Why? Because F_1_–F_n_ and F*_1_–F*_m_ both contribute to the same the property or capacity of removing corks, G. It should be emphasized that, consistent with his Dimensioned approach to realization, Gillett does not say that the two *corkscrews* are multiple realizations of the property of being a corkscrew. Instead, he says that the distinct property instances, F_1_–F_n_ ≠ F*_1_–F*_m_, of the aluminum and steel are distinct realizations.

Gillett further illustrates the view with a case in which other properties/relations of some of the constituent atoms does *not* lead to multiple realization. He writes,

Do proponents of the dimensioned metaphysics … take all differences of composition to be instances of multiple realization? To see that they do not, consider two aluminum corkscrews that are similar in all other respects except that one is made of aluminum containing a trace element. This element does not chemically bond with the aluminum, or change the metallic structure of aluminum atoms, but it does absorb a certain wavelength of light giving this corkscrew a yellow tinge. The same structure of aluminum atoms is therefore responsible for rigidity in both corkscrews, but there is a trace element in one of them (Gillett, 2003, pp. 598–599).

Gillett’s point is that the properties/relations of the atoms of the trace element do not contribute to the second corkscrew’s property of/capacity for removing corks, hence that the properties/relations of the atoms of the trace element do not realize the second corkscrew’s property of/capacity for removing corks. Thus, the properties/relations of the atoms of these two corkscrews represent only one realization of corkscrew. It should again be emphasized that, consistent with his Dimensioned approach to realization, Gillett does not say that the two *corkscrews* are a single realization of the property of being a corkscrew. Instead, he says that the numerically distinct properties/relations of the constituent aluminum atoms provide for a single realization of the property of being a corkscrew.

As a separate objection, Polger and Shapiro comment that “It would be odd indeed if the autonomy of psychology from neuroscience could be secured in virtue of tiny differences in potassium atoms” [Polger and Shapiro (2016), p. 39]. This, however, is not the Aizawa-Gillett view. Aizawa and Gillett (2009a) proposed that claims of realization and multiple realization are always indexed to particular levels and specific properties at these levels.

We can quickly see the importance of this point. Suppose that some higher level property G is multiply realized by microphysical properties of fundamental particles and hence multiply realized at the microphysical level. This does not, of course, mean that G is multiply realized in, say, distinct physiological properties (Aizawa and Gillett, 2009a, p. 550).

Applying what Aizawa and Gillett write, one does not get the multiple realization (or autonomy) of psychology from neuroscience by appealing to chemical properties of potassium.

Polger and Shapiro further object that the Aizawa-Gillett approach “entails an undesirable profligacy of distinct realizations for every kind, and undermines the significance of realization within debates over the autonomy of the special sciences” [Polger and Shapiro (2016), p. 39].^15^ Aizawa and Gillett (2009a) propose to develop a theory of realization and multiple realization that is meant to characterize compositional relations in the sciences. Once one has this theory, it is to a first approximation an empirical matter just how much multiple realization there is in the world. One should not judge *a priori* that a form of multiple realization is, or is not, pervasive.

We should perhaps go beyond what Aizawa and Gillett have already written to consider a confusion that seems to underlie Polger and Shapiro’s reasoning. Polger and Shapiro do not distinguish two claims. On the one hand, there is the claim that Dimensioned multiple realization is pervasive and, on the other, there is the claim that Dimensioned multiple realization is in some sense unimportant or trivial. Aizawa and Gillett believe that multiple realization is a pervasive feature of the biological world. They explicitly endorse, for example, the massive multiple realization of psychological properties.^16^ But, what is the connection between Dimensioned multiple realization being pervasive and Dimensioned multiple realization being trivial or undermining the significance of realization? Polger and Shapiro do not say. They appear not to see the difference between these claims, so perceive no need for an argument.^17^ Take an analogy to illustrate the point. Sexual reproduction is a pervasive feature of the biological world but is it not a trivial feature of the world. It is a kind of serious fact about life on earth that evolutionary biologists are very much concerned to understand and explain. Similarly, Aizawa and Gillett take pervasive Dimensioned multiple realization to be a serious fact about the world that merits philosophical attention.

## Critiques of the Aizawa-Gillett application

### The Polger and Shapiro critique

What reasons do Polger and Shapiro give to challenge the application of Aizawa and Gillett’s theory to the science of human color vision? They begin by switching from the property of having normal color vision to the property of trichromacy. They, then, caution that trichromacy might be a behavior or a behavioral capacity. They write,

To say that human beings are trichromats or that normal human color vision is trichromatic is to say that normal human beings exhibit a certain behavioral pattern. Trichromacy is the capacity to do a certain task—to match a sample using three primary lights. “Being trichromatic” is more like “being graceful” than it is like “being a vertebrate”; it is a behavior or effect that might have many causes. This makes us hesitant about whether the example of “normal human color vision” is an example of an internal or cognitive process at all, rather than the output of such a process [Polger and Shapiro (2016), p. 107].

This “cautionary note” is entirely misplaced. There are two distinct claims here. First, that trichromacy is a behavior or a behavioral pattern and, second, that it is a behavioral capacity. Let us consider these in order.

It is unclear why they think trichromacy is a behavior or a behavioral pattern. An individual might be a trichromat even in the dark or while sleeping. An individual is not a trichromat at just the time that individual is performing a matching test. Many, perhaps most, individuals who are trichromats never take such tests. Surely almost all the non-human primates that are trichromats never take such tests. As for the idea of a behavioral capacity, one can fathom how they got this idea. Earlier in their discussion they comment, “Normal human color vision is trichromatic, meaning that normally sighted human beings can match almost any color sample by mixing three different “primary” lights (Surridge et al., 2003).’’ So, let us concede for the sake of argument that there is a trichromatic behavioral capacity which is a capacity to successfully match. We might then ask how an agent has this behavioral capacity. Presumably the agent has the behavioral capacity in virtue, in part, of some visual perceptual capacity. In the typical case, if the agent did not have the visual perceptual capacity, the agent would not have the behavioral capacity. The picture here is the quite familiar one in cognitive science in which behavioral capacities depend on a lot of other capacities, many perceptual and cognitive, acting together. One of the core contentions of the cognitive revolution was that a behavioral capacity to speak a natural language involves a psychological linguistic capacity. The point is that even if there is a trichromatic behavioral capacity that does not show that there is not also a trichromatic visual perceptual capacity. Indeed, in typical cases, the latter would seem to be required for the former. Polger and Shapiro say nothing to undermine this familiar picture.^18^

### The Balari and Lorenzo critique

Consider, next, what Balari and Lorenzo have to say about the science on which Aizawa and Gillett rely. The drift of their critique is that Aizawa and Gillett ignore scientific practice, because Aizawa and Gillett do not use homology as a standard for similarity and difference. Balari and Lorenzo focus on Aizawa and Gillett’s discussion of the biochemistry of memory consolidation, but their discussion could apply just as easily to the biochemistry of human color vision. Here is the crucial passage

“[e]ven homologous proteins will differ to a greater or lesser degree in their amino acid sequences, so that they will differ to a greater or lesser degree in their physico-chemical properties” (Aizawa and Gillett, 2009b, p. 200). These words are illustrative, because they suggest that Aizawa and Gillett are here disregarding what counts as the same or different in the sciences, in molecular biology in this case. They appear not to be at all impressed by the fact that biologists and biochemists consider these proteins homologous and have developed their methods to determine homologies at this (and other) levels (Balari and Lorenzo, 2019, p. 18).

Notice the two dramatically different claims in this passage. The first is the claim that Aizawa and Gillett disregard what counts as the same or different in the sciences. The second is, in essence, the claim that Aizawa and Gillett disregard what counts as the same or different in terms of homology. The first would be problematic, if true. But it is false. The second is true but is unproblematic. There is a reason that Aizawa and Gillett do not adopt the criterion of homology, namely, there are other scientific standards of similarity and difference, those standards are the ones that are used in the portion of vision science under examination, and that is the science that is relevant for understanding the compositional relations in science.

Consider, first, the biochemistry of memory consolidation.^19^ In outline, the Aizawa-Gillett claim is that memory consolidation, G, is *probably* multiply realized by one set of property instances, F_1_–F_n_, in mice, another set of property instances, F*_1_–F*_m_, in *Drosophila*, and another set of property instances, F^**^_1_–F^**^_l_, in *Aplysia*. The argument for this begins with the observation that biochemists have identified distinct proteins, i.e., distinct chains of amino acids, in each of these species. Aizawa (2007), cites scientific work by Bartsch et al. (1998), Bergold et al. (1992), Beushausen et al. (1988), Kalderon and Rubin (1998), and Yin et al. (1994), in support of this view. They next proposed that differences in amino acid sequences are likely to generate differences in the properties of the proteins, thus, *probably* yielding multiple realization. Clearly, scientists distinguish proteins in terms of their amino acid sequences and distinguish them in terms of the properties, such as their binding constants, that they contribute to memory consolidation. So, it is clearly false to say that Aizawa and Gillett disregard what counts as the same or different in the sciences. The science was previously set out in Aizawa (2007).

Return now to the science of human color vision. In the memory consolidation case, there was an inference from differences in amino acid sequence to a difference in property instances that realize memory consolidation. The experimental work cited did not include direct measurements of, for example, the binding constants of the different proteins involved in LTP.^20^ Thus, there was, in point of logic, some room for empirical doubt. That was the basis of the italicized qualifier *probably*. The human color vision case addresses that source of empirical doubt. In the human color vision case, vision scientists know both the amino acid sequences and the absorption spectra of the cone opsins. Further, vision scientists know that two individuals with normal color vision can differ in the absorption spectra of their cone opsins. In support of this, Aizawa (2018b, cites Winderickx et al., 1992; Neitz and Neitz, 1998; Sjoberg et al., 1998; Sharpe et al., 1999). Balari and Lorenzo do nothing to square Aizawa and Gillett’s use of these scientific facts with the idea that they fail to respect scientific practice. Surely the charges of ignoring scientific facts must be dismissed.

Balari and Lorenzo are correct in noting that Aizawa and Gillett focus on, for example, whether two cone opsins have the same or different absorption spectra, but not on whether two cone opsins are homologous. One reason is that even if one accepts the need for identity criteria based on homology, one also needs identity criteria that are not so based. Clearly scientists recognize that distinct amino acid sequences have distinct properties, such as their absorption spectra.

What explains the connection between distinct amino acid sequences and distinct absorption spectra? Aizawa and Gillett (2019) propose that scientists give Standing Compositional explanations of such things. The absorption spectrum of a given amino acid chain is scientifically explained in terms of the individual amino acids of that chain, their primary sequence, and individual property instances. Scientists have this basic picture—though the complexity of the case makes it typically practically impossible—and Aizawa and Gillett offer a theory of this scientific picture.^21^

### The Strappini et al., critique

Like Polger and Shapiro and Balari and Lorenzo, Strappini et al., have doubts about the extent to which the human color vision case illustrates multiple realization. Here is the bulk of their critique,

Somehow in line with Polger and Shapiro (2016), we think that in the example provided by Aizawa and Gillet [*sic*], the cognitive property is missing. We do not exclude *a priori* that color perception (or being trichromat) can be considered a psychological property; however, we think that its phenomenology, its behavioral outcome, is missing from the proposal. We further conjecture that this example could provide concrete evidence of multiple realization if the psychological level was added by showing that there are no differences in color perception among trichromats that have those polymorphisms. Indeed, even slight differences among these normal trichromats would exclude that color vision is multiply realized (Strappini et al., 2020, p. 8).

To begin with, there are unclarities in what Strappini et al., are saying in the first part of this passage. What is this “cognitive property” they have in mind. And, “phenomenology” is often understood to be a kind of subjective feel, rather than a behavioral outcome. That, however, does not seem to be the core of their objection. Instead, their substantive claim is that there must be no differences in color perception between individuals with, say, Red(ala^180^) and Red(Ser^180^).

There is a long-standing idea that multiple realization requires, at the least, that the realizers be distinct and that the realized must be the same. Aizawa and Gillett’s application is meant to respect this. Indeed, it does so in three ways. First, Aizawa and Gillett propose that ‘‘normal color vision’’ as scientists use it in this context focuses on one property, but excludes certain other properties that one might lump under a pedestrian concept of normal color vision or of other scientific conceptions that might be labeled ‘‘normal color vision.’’ It focuses on the ability to make certain visual color discriminations. It excludes, for example, rapidity of response, luminance sensitivity, etc.^22^ So, there are some differences in color perception that are not included in the concept of “normal color vision” that are in play in this example.

Second, Aizawa and Gillett note that normal color vision, as used in the context, is a property that individual humans may have, even though there are individual differences in color discrimination among those who have this property. The Ishihara test, for example, is widely accepted as screening for normal human color vision, but a more sensitive Rayleigh color matching test is able to detect color matching differences among individuals with distinct cone opsins.^23^ Thus, there is a constant property that persists in the face of individual diversity.

Third, there is Aizawa and Gillett’s example of transducin. The crucial points of the example are that (1) property instances of individual transducin molecules realize normal color vision and (2) differences in these property instances do not induce individual differences in normal human color discriminations. See section ‘‘Critiques of the Aizawa-Gillett theories’’ above.^24^ Thus, the case cannot be dismissed on the grounds that the differences in transducin property instances merely induce individual differences.^25^

One reviewer has proposed that some further clarifications are required to address Strappini et al.’s objections:

When presenting the Aizawa-Gillett approach, the author adopts a theory of properties according to which properties are individualized by their causal powers. It seems plausible that the property of normal color vision is, *inter alia*, individualized by powers related to abilities for discriminating between colors. However, as suggested by Strappini et al., people with different absorption spectra of retinal cone opsins differ in abilities for color discrimination. It suggests that normal color vision is not, in fact, a single property, but rather a set of similar properties such that people with different absorption spectra possess different properties from this set. In this case, normal color vision is not a good example of multiply realized property.

The core of the “suggestion” here is that there is no property of normal human color vision, so no property to be multiple realized.

There is a lot to be said to address this, but much of it takes us far afield of the science of human color vision. To begin with, Strappini et al., do not give the foregoing argument. Although they do mention that Aizawa and Gillet are committed to the causal theory of properties, they do not use it as part of an objection to the proposal that there is a property of normal color vision. The closest they come is saying that “We do not exclude *a priori* that color perception (or being trichromat) can be considered a psychological property” (Strappini et al., 2020, p. 8). Second, what reason is there to think that there is no property of human color vision detected by, for example, the Ishihara test, but that there are other properties that are detected by, for example, Rayleigh matching?

The reviewer’s proposal invites emphasizing the importance of the role of transducin G-protein in human color vision. Suppose, simply for the sake of argument, that there is no property of normal color vision just as the reviewer proposes. Instead, there are only the “fine grained” properties of individual color discriminations as might be detected through Rayleigh matching. Even those very fine color discriminations will remain the same in the face of differences in the binding properties of transducin. One would not have multiple realization of normal human color vision by different cone opsins, but one would have multiple realization of “fine color discriminations” by instances of the binding constants of transducin. This story bears a lot more attention than it has so far received.

## Conclusion

For the last 25 years or so, a group of philosophers of science have tried to resolve questions of realization and multiple realization by closer attention to scientific practice. Aizawa and Gillett have long been a part of this. Over many years, they have developed a detailed theory of realization and multiple realization that is part of a broader account of compositional relations and compositional explanations in the special sciences. Further, they have provided numerous detailed case studies intended to illustrate its ability to account for actual scientific theorizing. The goal of this paper has been to draw together some of principal features of their work to show how the Aizawa-Gillett package of ideas addresses some of the objections that have appeared in the literature.

## Author contributions

The author confirms being the sole contributor of this work and has approved it for publication.

## Footnotes

1 For a contrary assessment, see Aizawa (2009).

2 For a contrary assessment, see Aizawa (2007).

3 See, for example, Rosenberg (2001) and Balari and Lorenzo (2015, 2019).

4 See, for example, Batterman (2000), Fang (2018, 2020), and Koskinen (2019).

5 Strappini et al. (2020, p. 8).

6 Gillett (2002, 2003) and Aizawa and Gillett, 2009a,b, 2011

7 For a discussion that places this species of explanation alongside others, see Aizawa and Gillett (2019).

8 One important feature this schema understates is how the powers of realized and realizer properties may be qualitatively distinct (see Gillett, 2002).

9 Balari and Lorenzo (2019), make a cryptic claim about the schema for Dimensioned realization. They write, Dimensioned realization “does not really go beyond telling us when and that (multiple) realization occurs, but falls short when it comes to explaining why or how some asymmetric dependence exists between entities at different levels” (Balari and Lorenzo, 2019, p. 5). The schema clearly states that asymmetric dependency between the property instances at one level, F_1_-F_n_, and the property instance at another level G arises when F_1_-F_n_ confers properties that are individuative of G. It is the conferring of properties that explains what Balari and Lorenzo ask for. (See also the discussion of Gillett, 2003 in section 2.0 below.) What is missing?

10 As an aside, the dipole moment example fares well in comparison to Polger and Shapiro’s favorite example of corkscrews. Corkscrews are artifacts. Moreover, the example does not fit Polger and Shapiro’s “Official Recipe”. See Aizawa (2020), but also Polger and Shapiro (2016, p. 67). Polger and Shapiro do not try to fit the corkscrew example into the schema. Instead, they merely gesture at what the fourth condition is supposed to do.

11 See, for example Merbs and Nathans (1992).

12 For further details, see Aizawa and Gillett (2011), section 10.3.3.

13 For a review and entry into this literature (see, e.g., Weinstein et al., 2006). For some reason, Aizawa and Gillett (2011), did not reference this literature.

14 Cf., e.g., Polger and Shapiro (2016, pp. 66f) and Strappini et al. (2020, p. 8).

15 Cf., Balari and Lorenzo (2015, p. 883).

16 Aizawa and Gillett (2009a, p. 540).

17 Balari and Lorenzo seem to be making essentially the same mistake in this passage: “assuming a criterion of identity that imposes strict equivalence of form or shape of biological structures, then, given the fact that inter- and intraspecific variation are the norm rather than the exception, multiple realization of any property will be trivially (and vacuously) true.” (Balari and Lorenzo, 2015, p. 883).

18 Polger and Shapiro argue that, on their theory of multiple realization, trichromacy is not multiply realized. Strictly speaking, this does not amount to a criticism of the Aizawa-Gillett approach. Readers might, however, want to have some idea of what to make of this. The simplest point is that the Polger-Shapiro view, being a flat view of realization, simply does not fit a Dimensioned case, like that of cone opsins. Consider their claim “(ii): There is a taxonomic system that distinguishes among trichromats—it sorts them by their peak sensitivities, say” [Polger and Shapiro (2016), p. 109]. (ii) is incorrect, as vision science does not distinguish among human trichromats in terms of their peak sensitivities. It distinguishes between cone opsins in terms of their peak sensitivities.

19 Here we aim for brevity. For more details, consult (Aizawa, 2007; Aizawa and Gillett, 2009a).

20 Recall the discussion of transducin above.

21 For further explanation of the work the Aizawa-Gillett theory might do for philosophy on this score, see (Aizawa, 2020).

22 For a more detailed exposition and defense of this point, see (Aizawa and Gillett, 2011, p. 211).

23 Cf., Aizawa and Gillett (2011, pp. 213–214).

24 See also Aizawa and Gillett (2011, section 10.3.3).

25 For the record, Polger and Shapiro (2016) and Strappini et al., seem to think that it is some sort of theory-neutral philosophical “datum” that individual differences cannot or should not give rise to multiple realization. In truth, this assumption is a consequence of Polger and Shapiro’s take on realization as a matter of an individual being a member of a kind. In other words, it is “theory-laden” presupposition.
